# Success and failure after surgery of degenerative disease of the lumbar spine: an operational definition based on satisfaction, pain, and disability from a prospective cohort

**DOI:** 10.1186/s12891-022-05460-0

**Published:** 2022-05-27

**Authors:** Albert V B Brasil, Maiara Anschau Floriani, Ericson Sfreddo, Tobias Ludwig do Nascimento, Andriele Abreu Castro, Luana Giongo Pedrotti, Marina Bessel, Juçara Gasparetto Maccari, Mohamed Parrini Mutlaq, Luiz Antonio Nasi

**Affiliations:** 1 Hospital Moinhos de Vento, Porto Alegre, Rio Grande do Sul Brazil; 2 Department of Neurosurgery, Grupo Hospitalar Conceição, Porto Alegre, Brazil; 3 Value Management Office (VMO), Grupo Hospitalar Conceição, Porto Alegre, Brazil; 4 Hospital Cristo Redentor, Grupo Hospitalar Conceição, Porto Alegre, Brazil

**Keywords:** Lumbar spine surgery, Real world evidence, Failure, Value-based health care, PROMS, Patient-reported

## Abstract

**Background:**

To describe success and failure (S&F) after lumbar spine surgery in terms equally understandable across the entire health ecosystem.

**Methods:**

Back and leg pain and disability were prospectively recorded before and up to 12 months after the procedure. Satisfaction was recorded using a Likert scale. Initially, patients were classified as satisfied or unsatisfied. Optimal satisfaction/unsatisfaction cutoff values for disability and pain were estimated with ROC curves. Satisfied and unsatisfied groups underwent a second subdivision into four subcategories: success (satisfied AND pain and disability concordant with cutoff values), incomplete success (satisfied AND pain and disability nonconformant with cutoff values), incomplete failure (unsatisfied AND pain and disability nonconformant with cutoff values), and failure (unsatisfied AND pain and disability concordant with cutoff values).

**Results:**

A total of 486 consecutive patients were recruited from 2019–2021. The mean values of preoperative PROMs were ODI 42.2 (+ 16.4), NPRS back 6.6 (+ 2.6) and NPRS leg 6.2 points (+ 2.9). Of the total, 80.7% were classified as satisfied, and 19.3% were classified as unsatisfactory. The optimal disability and pain cutoff values for satisfaction/unsatisfaction (NPRS = 6, AND ODI = 27) defined a subdivision: 59.6% were classified as success, 20.4% as incomplete success, 7.1% as incomplete failure and 12.4% as failure. The descriptions of each group were translated to the following: success—all patients were satisfied and presented no or only mild to tolerable pain and no or borderline disability; incomplete success – all patients were satisfied despite levels of pain and/or disability worse than ideal for success; incomplete failure – all patients were not satisfied despite levels of pain and/or disability better than expected for failure; failure – all patients were unsatisfied and presented moderate to severe pain and disability.

**Conclusion:**

It is possible to report S&F after surgery for DDL with precise and meaningful operational definitions focused on the experience of the patient.

## Background

The surgical treatment of degenerative disk disease of the lumbar spine (DDL) is characterized by the heterogeneity of indications, techniques and results [1]. At the same time, this type of surgery is accompanied by the greatest failure rate among the main surgeries of the locomotor system [2]. In the process of shared decision-making, doctors have the obligation to inform patients of this fact [3], and patients have the right to receive this information. The content of this information should be the clinical pictures of success and failure (S&F) as well as their relative incidences.

The problem in contemporary medicine is that the concept of S&F has never been precisely defined [4–6]. However, an enormous number of decisions are made every day with the objective of avoiding failure or seeking success. These decisions are made not only by patients and doctors but also by payers, hospitals, and industry. To improve the interaction between all of these stakeholders, one requirement is obvious: when one says “failure” or “success”, everybody should understand the same thing.

This scenario offers an opportunity for the utilization of an operational definition. Operational definitions may not be perfect, but they allow an honest and predictable interaction among everybody involved in a process. A good operational definition must balance precision (that is, be based on relevant and well-measured data) and communicability (that is, be expressed in terms that can be understood and make sense to all). The a priori hypothesis of this study is that an operational definition of S&F after lumbar spine surgery that attends to these requirements may be formulated by the combination of satisfaction, pain, and disability measures.

## Methods

### Population and data collection

This study was performed at Hospital Moinhos de Vento (HMV), a private institution with 485 beds and limited medical staff. HMV has a clinical database for many diseases following the ICHOM criteria [7].

Cases of DDL, such as lumbar disk herniation, lumbar stenosis, degenerative spondylolisthesis and back pain due to disk degeneration treated surgically from May 2019 to February 2021 and followed up to 12 months are included in the database. Patient-reported outcome measures (PROMs) and data relative to the number of levels and surgical technique were also registered.

### Ethics and consent

The study was approved by the Ethics Committee of the Moinhos de Vento Hospital (number 4.543.282 CAAE 41,454,920.3.0000.5330), and only participants providing a completed informed consent form participated. Data from medical records and PROMs were collected after informed consent was obtained from the participants. All methods in the study were performed in accordance with relevant institutional and national guidelines.

### Patient-Reported Outcome Measures (PROMs)

PROMs questionnaires were administered by trained interviewers from the hospital`s value management office pre- and postprocedure (telephone and/or electronic forms), and satisfaction was measured using a Likert scale [8] at four levels in the postoperative period. Likert 1 and 2 – very satisfied or satisfied – was used to classify patients as “satisfied”, and Likert 3 and 4 – dissatisfied or very dissatisfied – was used to classify patients as “unsatisfied”.

Functional disability related to the lumbar spine was reported by the Oswestry Disability Index (ODI) [9]. Back and leg pain were measured by the Numerical Pain Rating Scale (0 to 10) [10] and identified as [NPRS back] [NPRS leg]. Quality of life was analyzed by the Euro Quality of Life 5-Dimension Scale (EQ5D) [11]. Inability to work and analgesic use were investigated preoperatively and at 6 and 12 months.

### Statistical analysis

Categorical variables were summarized using absolute frequencies and percentages, while continuous variables were analyzed using means, standard deviations, medians and interquartile ranges. To compare proportions, the chi-squared test and Fisher’s exact test were used when appropriate, and the Mann–Whitney U test was used to compare continuous variables.

Analyses were stratified into pre- and postoperative periods. Subsequently, the postoperative group was divided into satisfied and unsatisfied groups, and comparisons between groups were performed. Furthermore, the postoperative group was also divided into success, incomplete success, incomplete failure and failure, and comparisons between group pairs were developed.

The optimal cutoff values of disability and pain were estimated by the receiver operating characteristic (ROC) curve by minimizing the Euclidean distance between the curve and the point (0.1) in the ROC space. The ROC curve of pain was built considering the highest value between NPRS back and NPRS leg. Areas under the curve (AUCs) and respective 95% confidence intervals (95% CIs) were also estimated. Sensitivity (Sen), specificity (Spe) and correct classification rate were also calculated for both measures. All analyses were performed using R software, version 4.0.3. Statistical significance was defined as a *p* value < 0.05.

## Results

During the study period, 486 patients underwent surgery, but 80 (16.4%) of the initial cohort did not respond to follow-up. After exclusion, the clinical cohort included 406 patients. Responders and nonresponders had similar background information (Table 1). Fifty-one surgeons participated in the study, but the majority of patients (343 pts – 84.5%) were operated on by 21 surgeons. The median age was 49.2 years [40.1–60.4], and 50.9% were male with a mean body mass index of 27.4 points. The education level was relatively high for the Brazilian population, with 56.1% having a university degree. The main comorbidities were hypertension (50.9%) and depression (10.8%), and 7.9% were smokers. The surgical techniques used were decompression with fusion (36.6%), simple decompression (31.1%) and automated percutaneous discectomy (26.9%) [12, 13]. Approximately one-quarter (24.4%) of the patients had a history of previous back surgery. All outcome measures showed improvement after the surgery.

**Table 1:** Baseline characteristics of the patients

	**n (%) or median [IQR]** **responders *n* = 406**	**n (%) or median [IQR]** **non-responders *n* = 80**	***P* value**
**DEMOGRAPHICS**			
Male	207 (50.9)	36 (45.0)	0.34
Age (y)	49.2 [40.1–60.4]	49.2 [37.2–59.9]	0.22
Caucasian	382 (94.0)	76 (95.0)	0.73
Tertiary education	228 (56.1)	44 (55.0)	0.86
Body mass index (BMI)	27.4 [24.9–30.4]	27.5 [25.1–30.5]	0.70
Smoke	32 (7.9)	9 (11.2)	0.33
COMORBIDITY			
Hypertension	112 (27.5)	19 (24.0)	0.52
Depression	44 (10.8)	1 (1.3)	< 0.01
Diabetes	25 (6.1)	7 (8.8)	0.37
Heart disease (heart failure, heart attack, angina, atrial fibrillation)	25 (6.1)	4 (5.0)	0.70
Pulmonary disease (emphysema, COPD, asthma)	24 (5.9)	4 (5.0)	0.75
Arthritis	23 (5.6)	1 (1.3)	0.10
Leg pain (poor circulation)	20 (4.9)	2 (2.5)	0.34
Cancer (last 5 y)	11 (2.7)	0 (0.0)	-
Liver disease	10 (2.4)	0 (0.0)	-
Chronic kidney disease	5 (1.2)	0 (0.0)	-
**CLINICAL INFORMATION**			
Mild systemic disease (ASA II)	271 (67.4)	50 (62.5)	0.40
History of spine surgery	99 (24.4)	24 (30.0)	0.29
Surgery classification			
*Decompression and arthrodesis*	148 (36.6)	28 (35.0)	0.79
*Automated percutaneous*	126 (26.9)	20 (25.0)	0.73
*Decompression*	109 (31.1)	9 (11.3)	< 0.01
Procedure level			
*1 or 2 levels*	314 (78.9)	65 (83.3)	0.37
≥ *3 levels*	84 (21.1)	13 (16.7)	0.37
Length of stay			
	2.0 [1.0–4.0]	1.0 [1.0–3.0]	0.38

Global pre- and postoperative outcomes are presented in Table 2. The preoperative ODI improved from 42.0 points [32.0–54.0] to 16.0 points [4.4–34.0] postoperatively. Back pain (NPRS back) and leg pain (NPRS leg) varied from 7.0 [5.2–8.0] and 7.0 [5.0–8.0] points to 3.0 [0.0–6.0] and 3.0 [0.0–6.0], respectively. The regular utilization of opioids decreased from 40.0% in the preoperative period to 19.2% in the postoperative period (*p* < 0.01). The reduction in the percentage of patients unable to work due to back pain (22.6% to 19.40% *p* = 0.26) was not significant.

**Table 2:** Pre- and postprocedure groups evaluated for pain, disability and quality of life

	**median [IQR] or mean (SD)**		***P* value**
	**Preprocedure**	**Postprocedure**	
**Oswestry Disability Index (ODI)**			
	42.0 [32.0–54.0]	16.0 [4.4–34.0]	< 0.01
	42.2 (16.4)	21.5 (19.7)	
**Numeric Pain Rating Scale (NPRS)**			
**NPRS low back**	7.0 [5.2–8.0]	3.0 [0.0–6.0]	< 0.01
	6.6 (2.6)	3.5 (3.1)	
**NPRS leg**			
	7.0 [5.0–8.0]	0.0 [0.0–5.0]	< 0.01
	6.2 (2.9)	2.3 (3.1)	
**EQ5D-3L**			
	0.731 [0.59–0.73]	0.787 [0.64–1.0]	< 0.01
	0.65 (0.10)	0.78 (0.20)	
**Prescription opioids**	**n (%)**		
* Not or sometimes*	243 (60.0)	328 (80.7)	< 0.01
* Regularly*	162 (40.0)	78 (19.2)	
**Over-the-counter analgesics**			
* Not or sometimes*	356 (87.9)	391 (96.3)	< 0.01
* Regularly*	49 (12.1)	15 (3.6)	
**Unable to work due to pain**	92 (22.6)	79 (19.4)	0.26
**Return to work (< 3 months)**		179 (81.7)	

### Satisfied and unsatisfied patients

The outcomes of satisfied (80.7%) and unsatisfied (19.3%) patients are presented in Table 3, and the clinical profile in the postoperative subgroups differed considerably. The satisfied group presented mean values of NPRS back = 2.0 [0.0–5.0], NPRS leg = 0.0 [0.0–4.0] and mean ODI = 12.0 [4.0–26.0] points. Unsatisfied patients presented mean values of NPRS back = 7 [0.0–8.0], NPRS leg = 4.0 [0.0–8.0] and mean ODI = 38.0 [24.0–52.0]. Significant improvement between the preprocedure and postprocedure values in satisfied group was observed in practically all parameters. On the other hand, almost no difference was present between the preprocedure and postprocedure values in unsatisfied group.

**Table 3:** Satisfied and unsatisfied groups evaluated for pain, disability and quality of life

	**Pre procedure (n 406)**	**median [IQR] or mean (SD)**		***P* value**
		**Satisfied (n 328)**	**Unsatisfied (n 78)**	
**Oswestry Disability Index (ODI)**				
	42.0 [32.0–54.0]	12.0 [4.0–26.0]	38.0 [24.0–52.0]	< 0.01^ab^ 0.06.^c^
	42.2 (16.4)	17.5 (17.4)	38.3 (19.8)	
**Numeric Pain Rating Scale (NPRS)**				
**NPRS low back**	7.0 [5.2–8.0]	2.0 [0.0–5.0]	7.0 [0.0–8.0]	< 0.01^ab^ 0.13.^c^
	6.6 (2.6)	2.8 (2.8)	6.1 (2.8)	
**NPRS leg**				
	7.0 [5.0–8.0]	0.0 [0.0–4.0]	4.0 [0.0–7.0]	< 0.01.^abc^
	6.2 (2.9)	1.9 (2.8)	4.1 (3.7)	
**EQ5D-3L**				
	0.73 [0.59–0.73]	0.79 [0.69–1.0]	0.66 [0.52–0.78]	< 0.01^ab^ 0.17.^c^
	0.65 (0.10)	0.81 (0.18)	0.63 (0.18)	
**Prescription opioids**		**n (%)**		
*** Regularly***	162 (40.0)	48 (14.6)	30 (38.5)	< 0.01^ab^ 0.80.^c^
**Over-the-counter analgesics**				
*** Regularly***	49 (12.1)	9 (2.7)	6 (7.7)	0.03^a^ < 0.01^b^ 0.26.^c^
**Unable to work due to pain**	92 (22.7)	56 (17.1)	23 (29.5)	0.01^a^0.06^b^ 0.20.^c^
**Return to work (< 3 months)**		156 (87.1)	23 (12.8)	< 0.01^a^

^a^compared satisfied and unsatisfied group ^b^compared pre-operative and satisfied.^c^compared pre-operative and unsatisfied

### Cutoff values of disability and pain according to satisfaction/unsatisfaction

**Fig. 1:**
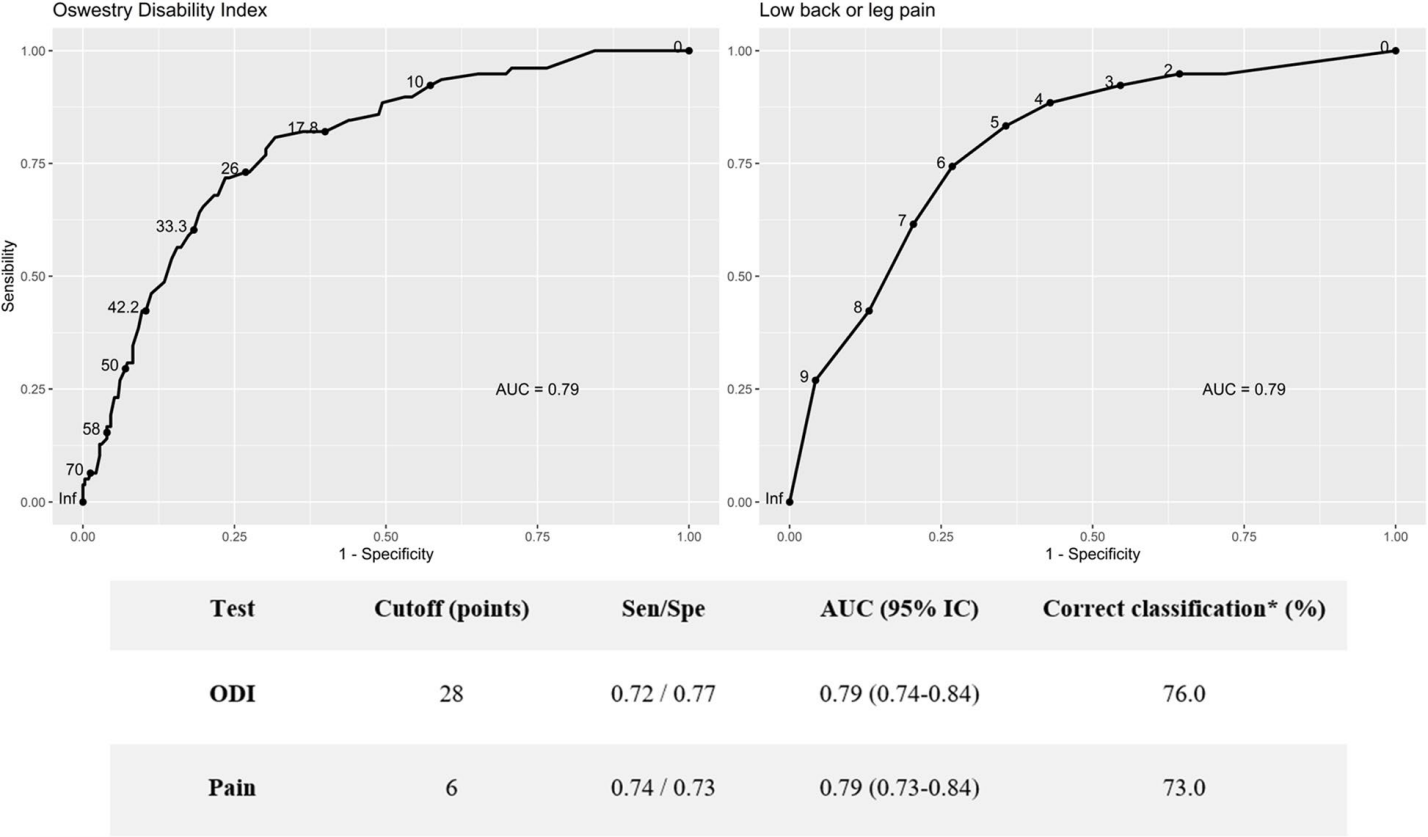
– Sensibility (Sen) and specificity (Spe) of ODI and pain values for S&F patients. *The correct classification rate is the sum of the number on the diagonal divided by the sample size in the test data

The sensitivity (Sen) and specificity (Spe) of the values of disability and pain used to discriminate between satisfied and unsatisfied patients were studied with ROC curves (Fig. 1), showing a narrow range of approximately 75.0% [72.0–77.0]. Both ROC curves [ODI (AUC 0.79) and pain (AUC 0.79)] presented an “ACCEPTABLE” performance (between 70.0 and 80.0) with values close to “GOOD” [14]. The cutoff values for ODI and back/leg pain were 28 and 6, respectively.

The data show that approximately 75.0% of satisfied patients presented pain ≤ 5, and 75.0% of unsatisfied patients presented pain ≥ 6 points. At the same time, ~ 75.0% of satisfied patients presented an ODI ≤ 27, and ~ 75.0% of unsatisfied patients presented an ODI ≥ 28 points.

### Success, incomplete success, incomplete failure and failure

The satisfied and unsatisfied groups were further subdivided based on concordance or nonconcordance with the discrimination cutoff values:

1. Success (59.6%)—satisfied with pain and disability levels concordant (NPRS ≤ 5, AND ODI ≤ 27);
2. Incomplete success (20.4%)—satisfied with pain and disability levels nonconcordant (NPRS ≥ 6 AND/OR ODI ≥ 28);
3. Incomplete failure (7.1%)—unsatisfied with pain and disability levels nonconcordant (NPRS ≤ 5 AND/OR ODI ≤ 27);
4. Failure (12.4%)—unsatisfied with pain and disability levels concordant (NPRS ≥ 6 AND ODI ≥ 28).

The PROMs values of the four categories are presented in Table 4. There was a very significant improvement between preoperative (ODI 42.0 [32.0–54.0], NPRS back 7.0 [5.2–8.0], NPRS leg 7.0 [5.0–8.0]) and postoperative values in the success subgroup (ODI 8.0[2.0–16.0], NPRS back 1.0 [0.0–3.0], NPRS leg 0.0 [0.0–1.0]), but there was almost no difference between preoperative and postoperative values in the failure subgroup (ODI 44.4[38.0–54.0], NPRS back 7.0 [6.0–9.0], NPRS leg 7.0 [1.0–9.0]). The mean PROMs values of the incomplete success and incomplete failure subgroups lie in between these two extremes.

**Table 4:** Failure and success groups evaluated for pain, disability and quality of life

	**Pre procedure** **(n 406)**	**median [IQR] or mean (SD)**		**Incomplete failure** **(n 29)**	**Failure** **(n 49)**	***P* value**
		**Success** **(n 242)**	**Incomplete success** **(n 86)**			
**Oswestry Disability Index (ODI)**						
	42.0 [32.0–54.0]	8.0 [2.0–16.0]	40.0 [31.1–50.0]	20.0 [10.0–26.0]	44.4 [38.0–54.0]	< 0.001^a,c,d^ 0.02.^b^
	42.2 (16.4)	9.2 (8.1)	40.9 (15.3)	22.6 (18.8)	47.7 (13.7)	
**Numeric Pain Rating Scale (NPRS)**						
**NPRS low back**	7.0 [5.2–8.0]	1.0 [0.0 – 3.0]	6.0 [4.0–8.0]	5.0 [2.0–6.0]	7.0 [6.0–9.0]	< 0.001^a,c,d^ 0.07.^b^
	6.6 (2.6)	1.9 (2.2)	5.7 (2.7)	4.1 (2.8)	7.3 (2.1)	
**NPRS leg**						
	7.0 [5.0–8.0]	0.0 [0.0–1.0]	6.0 [3.0–7.8]	0 [0.0–4.0]	7.0 [1.0–9.0]	< 0.001^a,c,d^ 0.05.^b^
	6.2 (2.9)	0.9 (1.8)	5.0 (3.1)	2.3 (2.9)	5.3 (3.8)	
**EQ5D-3L**						
	0.73 [0.59–0.73]	1.0 [0.8–1.0]	0.6 [0.5–0.7]	0.7 [0.6–0.8]	0.5 [0.5 – 0.7]	< 0.001^a,b,c^ 0.04.^d^
	0.7 (0.1)	0.9 (0.1)	0.6 (0.2)	0.7 (0.2)	0.6 (0.2)	
**Prescription opioids**		**n (%)**				
*** Regularly***	162 (40.0)	42 (17.4)	60 (69.8)	8 (27.6)	36 (73.5)	< 0.001^a,b,c,d^
**Over-the-counter analgesics**						
*** Regularly***	49 (12.1)	3 (1.2)	6 (7.0)	0 (0.0)	6 (12.2)	< 0.001^a,c^ 0.98.^b^
**Unable to work due to pain**	92 (22.7)	21 (8.7)	35 (40.7)	6 (20.7)	17 (34.7)	< 0.001^a,c^ 0.06^b^ 0.19.^d^
**Return to work (< 3 months)**		130 (81.2)	26 (86.7)	13 (86.7)	10 (71.4)	0.25^c^ 012.^d^

^a^Pre procedure vs Success, ^b^Pre procedure vs Failure, ^c^Success vs Incomplete success, ^d^Failure vs Incomplete Failure

## Discussion

We measured satisfaction, pain, and disability in a cohort of 406 patients who underwent surgery for DDL. Based on the combination of PROMs, we created four outcome categories in the following terms: success (59.6%)—satisfied with pain and disability levels concordant (NPRS ≤ 5, AND ODI ≤ 27); incomplete success (20.4%)—satisfied with pain and disability levels nonconcordant (NPRS ≥ 6 AND/OR ODI ≥ 28); incomplete failure (7.1%)—unsatisfied with pain and disability levels nonconcordant (NPRS ≤ 5 AND/OR ODI ≤ 27); and failure (12.4%)—unsatisfied with pain and disability levels concordant (NPRS ≥ 6 AND ODI ≥ 28).

The clinical profile of success (ODI 8.0 [2.0–16.0], NPRS back 1.0 [0.0–3.0], NPRS leg 0.0 [0.0–1.0]) is comparable with the normal healthy population, that is, pain in the range of “no pain” [10] and ODI in the range of the healthy population [9]. At the same time, the clinical profile of failure (ODI 44.4 [38.0–54.0], NPRS back 7.0 [6.0–9.0], NPRS leg 7.0 [1.0–9.0]) demonstrates that these patients remain as sick as they were before surgery. This model seems well adjusted to the common ideas of success (suggestive of normal life) and failure (continuation or worsening of the disease).

It is intuitive that there is not a sharp limit between S&F. Intermediary categories were then created for satisfied patients with pain and disability worse than expected (incomplete success) and for unsatisfied patients with pain and disability better than expected (incomplete failure).

### Methodological issues

Our S&F model is based on satisfaction, disability, and pain, with satisfaction as the main criterion. The choice of satisfaction as the primary anchor may be debated. Some authors demonstrate that there is a discrepancy between satisfaction and PROMs [15], while others demonstrate that they correlate well [16]. It is clear that satisfaction correlates better with the final raw scores than with improvement [17]. It was hypothesized that PROMs may not be the best instrument for evaluating satisfaction [18] because satisfaction depends on a complex and wider array of variables, such as physical and mental health, expectations and lifestyle [16, 18]. Some authors chose satisfaction as the main translation of success [16] and were praised for that [19]. Even the concept of minimal clinically important difference (MCID) is based on satisfaction. Satisfaction represents the patient’s most comprehensive evaluation of what occurred [20].

We then chose ODI and NPRS [21] as complementary criteria because they are directly related to the disease. Quality of life is also important in this evaluation, but it is dependent on other social and health factors. EQ5D varies among countries and is difficult to explain in simple words. In the same manner, drug use and work status are also important but were left out of the model because they evaluate the consequences of the disease and not the disease itself.

For the method of this study, we adopted the final raw score of pain and disability as outcomes. Many authors base their studies on preoperative-to-postoperative variation as well as on MCID [22–24]. Previous studies demonstrated that the analysis S&F based on preoperative-to-postoperative differences or MCID may have some flaws [6]. The results obtained with this strategy are strongly influenced by the severity of preoperative symptoms [25, 26]. Final raw scores correlate better with S&F and are simpler and more objective, and they are not influenced by the intensity of preoperative symptoms [25]. Our model describes “how patients will be at the end of treatment” (final raw scores) and avoids referring to an elusive “minimum clinically significant” improvement.

Another peculiarity of our study was to assess pain considering the highest value between back and leg pain. We assume that the patient’s suffering is better assessed in this manner. Other authors have previously done the same [4].

### Translation of numerical values into simple and meaningful terms

The translation of numerical values into simple and meaningful terms is the aim of our study. It is not exactly a “result” because it was not originally extracted from our data. A summary of the available literature will be presented in this section to support our rationale.

Satisfaction was linked to back/leg pain ≤ 5 in our cohort as well as in previous similar studies [23, 27]. Pain scales can be numerical, visual or verbal, and the equivalence among these three forms has already been studied [10, 28]. For a numeric scale, no pain is represented by pain 0 to 2; 3 to 4 is described as mild pain; 6 to 8 is moderate pain and 9 to 10 is severe pain. From the verbal standpoint, pain = 5 is located exactly in the midpoint between mild and moderate pain. However, what is the best word to describe pain = 5?

Zelman and coworkers [29] studied the interference of pain in the life of chronic low back pain patients (sensation of controlled pain, ability to participate in productive activities, decreased irritability, low analgesic intake and willingness to socialize). In this analysis, it was demonstrated that a pain = 5 represented the limit between tolerable and intolerable pain. The cutoff value of 5 for back/leg pain was found by us and by other authors. Our data as well as those of the literature support the idea that “tolerable” is an appropriate term to describe pain = 5. According to this information, patients with pain ≤ 5 can be described as having no or only mild to tolerable pain.

In Japan [17], the mean ODI value of patients who were disabled due to spine problems varied from 26 and 28 points at the ages of 50 and 70 years, respectively. In other studies, the criteria were stricter, and the mean ODI was 21 points for success [25] and 25 points for failure [27]. Most studies based on final raw scores found cutoff values for failure between 22 and 30 points [23, 24, 30].

In the short term, the pertinent literature determines the existence of a borderline zone between the ODI values of disabled and nondisabled patients, ranging from 21 to 31 points. In our cohort, an ODI ≤ 27 points were linked to satisfaction, and this value lies within this borderline zone. Therefore, patients with an ODI ≤ 27 points can be described as individuals with no disability or borderline disability.

### Operational definitions

Our results support the description of four operational definitions:

1. **Success**– All patients are satisfied, and present no or only mild to tolerable pain and no or only borderline disability.
2. **Incomplete success** – All patients are satisfied despite levels of pain and/or disability worse than ideal for success.
3. **Incomplete failure** – All patients are not satisfied despite levels of pain and/or disability better than expected for failure.
4. **Failure –** All patients are unsatisfied, and all present moderate to severe pain and disability.

### The option for an operational definition of S&F

The precise concept (or diagnostic criteria) of S&F after low back surgery has never been and will probably never be defined [4–6]. Nonetheless, S&F happen and are widely studied. One review at PUBMED with the terms “lumbar spine surgery AND failure” showed 3,268 results. Another one with “lumbar spine surgery AND success” generated 2,882 results. Concepts or definitions of S&F are based on many PROMs that measure different constructs, so their results are almost never coincident [31]. As a result, patients face a myriad of numbers that are difficult to understand. According to some authors, even doctors have difficulty fully understanding the meaning of these numbers [27].

A process of shared decision based on concepts such as “33.0% improvement in ODI” or “to reach MCID in leg pain” is almost impossible. This difficulty is more visible in people with low literacy but can happen in more educated people [32]. According to Werner et al. [27], patients have a greater ability to understand the percentages of definite types of outcomes than continuous variables. Our method responds to these problems in two ways: a) it divides the possible outcomes into four intuitive types (success, incomplete success, incomplete failure and failure), and b) the myriad of numeric variables was replaced by simple equivalent words.

We emphasized the importance of our operational definitions for communication among all stakeholders of spine surgery. However, there is one specific scenario where this type of definition reaches its most relevant moment: this is the preoperative discussion between patient and doctor concerning the indication of surgery [33]. Patients have the right to be informed, and doctors must be in charge of giving the information concerning all possible outcomes, that is, their relative incidences and clinical characteristics. This information must be as precise as possible and be presented in simple and meaningful terms. This is a prerequisite to ensure that patients can exert their freedom of choice [34, 35].

### Possible deficiencies of the study

This study was based on a single institution, so our results need to be replicated and tested to obtain better validation. Our cohort included different diseases (disc herniation, stenosis, etc.), surgical techniques, approaches, and surgeons in one single group. This is in line with a recent tendency of the surgical literature, the so-called *science of practice* [36, 37]*.* With this approach, it was already demonstrated, for example, that return to work [38], improvement of pain, disability and quality of life depend more on the patients’ characteristics than on the type of approach, number of levels, use of fusion, surgeon’s experience and other factors [39].

Other criticisms can be made on the lack of attention to relevant clinical aspects such as the relatively short follow-up in patients who underwent fusion and the influence of educational level or previous surgery on the results. The objective of this study, however, is not to describe the rates of success and failure (which truly may depend on timing or many other variables) but rather to describe a manner (simple and communicable) of reporting the basic endpoints of success and failure. The rates of S&F may change, but the way they are described may not.

Finally, there is the problem of reducing all possible outcomes into only 4 categories. The complexity of degenerative disc disease and the heterogeneity of treatments and results deserve a very granular subdivision of possible outcomes. Such a “perfect” definition, on the other hand, would be cumbersome during the process of decision-making. It must be recognized that the broader aim of developing a completely truthful and sophisticated definition of S&F has not proven feasible in the context of lumbar spine surgery. The more complex and sophisticated the definition, the more difficult it is to be understood and communicated, and vice versa. This tradeoff is inevitable. It is the opinion of the authors that the simplicity and communicability of our operational definitions were obtained without compromising precision.

## Conclusion

It is possible to report S&F after surgery for DDL with operational definitions based on satisfaction, disability, and pain that are precise, simple, and meaningful to all people involved in the process. Our operational definitions of success, incomplete success, incomplete failure, and failure may improve the process of shared decisions focused on the experience of the patient.

## Data Availability

The datasets generated and/or analyzed during this study are not publicly available because they are patient data. The institution does not allow the sharing of raw data, but they are available from the corresponding author upon reasonable request.

## Abbreviations

DDL: Degenerative disk disease of the lumbar spine
EQ5D: Euro Quality of Life 5-Dimension Scale
MCID: Minimal clinically important difference
NPRS: Numerical Pain Rating Scale
ODI: Oswestry Disability Index
PROMs: Patient-reported outcome measures
